# Power and poverty: A participatory study on the complexities of HIV and intimate partner violence in an informal urban settlement in Nairobi, Kenya

**DOI:** 10.1016/j.socscimed.2023.116247

**Published:** 2023-11

**Authors:** Beate Ringwald, Miriam Taegtmeyer, Veronicah Mwania, Mary Muthoki, Faith Munyao, Lina Digolo, Lilian Otiso, Anne S. Wangui Ngunjiri, Robinson N. Karuga, Rachel Tolhurst, for the Korogocho ALIV[H]E research team

**Affiliations:** a ARISE Hub, Department of International Public Health, Liverpool School of Tropical Medicine, Pembroke Place, Liverpool, L3 5QA, UK; b Community Health Systems Group, Department of International Public Health, Liverpool School of Tropical Medicine, Pembroke Place, Liverpool, L3 5QA, UK; c LVCT Health, P.O. Box 19835-00202, KNH, Nairobi, Kenya; d The Prevention Collaborative, P.O. Box 3794 00100, Nairobi, Kenya

**Keywords:** HIV, Intimate partner violence, Informal urban settlement, Kenya, Sexual and reproductive health and rights, Gender equality, Participatory health research

## Abstract

People in informal urban settlements in Kenya face multiple inequalities, yet researchers investigate issues such as HIV or intimate partner violence (IPV) in isolation, targeting single populations and focusing on individual behaviour, without involving informal settlement dwellers. We formed a study team of researchers (n = 4) and lay investigators (n = 11) from an informal settlement in Nairobi, Kenya to understand the power dynamics in the informal urban settlement that influence vulnerability to IPV and HIV among women and men from key populations in this context. We facilitated participatory workshops with 56 women and 32 men from different marginalised groups and interviewed 10 key informants. We used a participatory data analysis approach. Our findings suggest the IPV and HIV nexus is rooted in the daily struggle for cash and survival in the informal urban settlement where lucrative livelihoods are scarce and a few gatekeepers regulate access to opportunities. Power is gendered and used to exercise control over people and resources. Common coping strategies applied to mitigate against the effects of poverty and powerlessness amplify vulnerabilities to HIV and IPV. These complex power relations create and sustain an environment conducive to IPV and HIV. Prevention interventions thus need to address underlying structural drivers, uphold human rights, create safe environments, and promote participation to maximise and sustain the positive effects of biomedical, behavioural, and empowerment strategies.

## Highlights

• A participatory study involving key populations from an informal urban settlement.
• Multi-layered power relations drive HIV and intimate partner violence (IPV).
• Economic and gender inequities fuel daily struggles for survival and vulnerability.
• Structural violence and coping strategies increase exposure to HIV and IPV more.
• Prevention strategies must address structural drivers, uphold rights and safety.

## 1 Introduction

Human immunodeficiency virus (HIV) and intimate partner violence (IPV) are linked (Li et al., 2014), undermining sexual and reproductive health and rights (Starrs et al., 2018). Women experiencing IPV are more likely to acquire HIV (Heise and McGrory, 2016), and women living with HIV are vulnerable to IPV, especially after HIV diagnosis and disclosure (Colombini et al., 2016; Orza et al., 2015). Similar intersections are observed among men who have sex with men (Buller et al., 2014). IPV-related vulnerability to HIV emerges through sexual violence, alcohol or substance use, condomless sex, and multiple sexual partnerships (Heise and McGrory, 2016). Partner conflicts around condom use and perceived infidelity can trigger IPV (Campbell et al., 2008). Men and women affected by HIV and IPV are less likely to access HIV care and have poorer treatment outcomes when they do (Hatcher et al., 2015; Schafer et al., 2012). HIV and IPV, affecting physical, mental, material, and social well-being, are global sexual and reproductive health and rights priorities (Starrs et al., 2018).

Power imbalances are key drivers of IPV and HIV. We define power as “*an ability to achieve a wanted end in a social context, with or without the consent of others*” (Vermeulen, 2005, p. 39) and as being relational and dynamic. Joanna Rowlands (1997) distinguished power as (1) control (or power-over) describing a finite quantity of power divided among actors to exercise control over others; and (2) a process whereby individuals discover their capacity (power-within), use their ability to act (power-to), and work together to achieve common goals (power-with). Power is constituted, exercised, contested, or resisted in visible, invisible, or hidden ways at different levels within society – from individual to global (VeneKlasen and Miller, 2007).

The ALIV[H]E framework promoting ‘action linking initiatives on violence against women and HIV everywhere’ builds on these power concepts (Salamander Trust et al., 2017). ALIV[H]E, which was developed through a multi-country participatory learning process, centres the expertise of women living with and affected by HIV (Hale et al., 2018). ALIV[H]E addresses internalised beliefs and behaviours as well as social norms and practices condoning men's use of violence against women. It also seeks to ease access to services and change discriminatory laws and policies towards sexual and gender minorities, sex workers, and people who inject drugs, who are criminalised, socially marginalised, and vulnerable to HIV and IPV.

Our understanding of intersectional power considers that social hierarchies related to gender, sexuality, age, and ability (among others) are interconnected and multiple power relations work together (Collins and Bilge, 2020; Hankivsky, 2014). It builds on intersectionality theory, developed in the USA to explain the nexus of gender and racial discrimination shaping Black women's experiences of violence (Crenshaw, 1991). It recognises the impact of colonialism on gender power relations and interconnected structural forces of power in African contexts (Steady, 2004).

This study was conducted in Kenya. The legacies of colonialism in Kenya, including the emergence of informal settlements in Nairobi, underlie current power dynamics (Omanga and Ndlovu-Gatsheni, 2020). Nairobi, founded as a railway depot by British officials in 1899 (Ogot and Ogot, 2020), housed 4.4 million people in 2019 (Kenya National Bureau of Statistics [KNBS], 2019). Colonial urban planning enforced racial segregation, excluding the African majority who lived in spontaneous settlements outside urban boundaries or later an overcrowded ‘native location’ (Ogot and Ogot, 2020). Nowadays more than half of Nairobi's population lives in crowded informal urban settlements, lacking durable housing, tenure security, clean water and sanitation (UN-Habitat, 2016).

Although the sex ratio of Nairobi's population has shifted from male to female majority (KNBS, 2019; Ogot and Ogot, 2020), gender power relations remain patriarchal. Men hold most elected positions in the county assembly (Kinyanjui, 2022). Women's economic opportunities have lagged; brewing alcohol and sex work were main sources of income in the past (Ogot and Ogot, 2020) and domestic service today (KNBS et al., 2015). Girls in informal urban settlements are faced with numerous barriers to education (African Population and Health Research Center [APHRC], 2014). Education and health services were segregated by race, and later by class (Ogot and Ogot, 2020). Social problems in informal urban settlements include unemployment, unmet family planning needs, teenage pregnancy (APHRC, 2014), and high burdens of HIV and IPV (Madise et al., 2012; Ringwald et al., 2022).

Despite existing knowledge on linkages between HIV and IPV, power and intersectionality, studies commonly investigate HIV or IPV in isolation; engage a single population such as young women; lack attention to structural factors influencing individual behaviour; and limit the involvement of affected populations (Mannell et al., 2019). We worked with women and men from key populations as co-researchers and together set out to gain a nuanced understanding of the power dynamics that influence IPV and HIV in the informal urban settlement.

## 2 Methods

We nested our study within ARISE, an international research consortium working in partnership with people in informal urban settlements. Kenyan ARISE partner, LVCT Health, a non-governmental organisation (NGO) specialising in HIV and gender-based violence (GBV), has established research partnerships within Nairobi’s informal settlement communities and implements DREAMS – an HIV prevention intervention for adolescent girls and young women targeting behavioural and structural factors, including GBV (Saul et al., 2018).

**Fig. 1:**
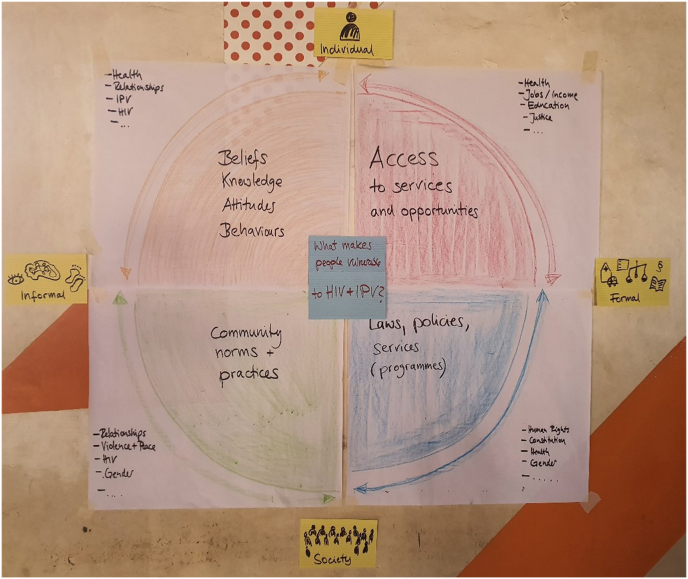
Conceptual framework. Power matrix adapted from ALIV[H]E (Salamander Trust, 2017).

Our conceptual framework for HIV and IPV linkages (Fig. 1), blending the ALIV[H]E power matrix with an intersectionality lens, enabled us to explore multiple interconnected layers of power and mitigate against monolithic representations of women and men in informal urban settlements (Ratele, 2008; Steady, 2004). ALIV[H]E and intersectionality link theory formation and use of knowledge by marginalised communities to challenge inequalities (Collins and Bilge, 2020; Salamander Trust, 2017). We operationalised these concepts through a participatory health research approach (Abma et al., 2019) and actively involved a diverse group of community co-researchers in the research process. Our jointly developed IPV definition refers to IPV as any behaviour within an intimate relationship causing emotional, physical, sexual, or economic harm. We considered intimate relationships to include heterosexual dating, cohabiting, marital, extra-marital, and transactional relationships, as well as romantic and transactional same-sex partnerships.

### 2.1 Study site

Our study was conducted in Korogocho, located 11 km North-East of Nairobi's city centre, near Nairobi's main rubbish dump and some industrial areas. Established in the 1960s, the settlement grew as the government resettled people from other parts of Nairobi. Korogocho's population (officially 37,000 in 2019) occupies 0.9 km^2^ – a sevenfold higher population density than Nairobi's average (KNBS, 2019). From a predominantly younger male population, the settlement has become an established community (Wamukoya et al., 2020).

People in Korogocho face multiple challenges. Most households (85%) live below the international poverty line (US$1.90/day) and reside in rented property (75%), mainly one-room structures with mud and timber walls, waste tin can roofs, and without piped water and flush toilets (Wamukoya et al., 2020). Female and male education levels lag behind Nairobi's average (APHRC, 2014). Many people rely on casual employment; unemployment is high, especially among women (42% vs 20% men) (Wamukoya et al., 2020).

Public institutions and NGOs provide various services in Korogocho. The Senior Chief, a national government officer supported by Assistant Chiefs and village elders, is tasked with maintaining order and preventing crime and oversees aid and development programmes (The Chiefs' Act of Kenya, 1937), including vetting beneficiaries (Sarnquist et al., 2018). Korogocho's Chief's camp accommodates the Senior Chief's office, community halls, a public health facility, community radio station, and NGOs. Designated policewomen operate the ‘GBV desk’ at Korogocho Police Station. At the time of this study, a public and some private (not-for-profit) health facilities (includingLVCT Health DREAMS site]) provided free HIV and gender-based violence (GBV) services to key populations and/or the public.

We selected Gitathuru, one of Korogocho's nine villages, for its ethnically diverse population and distinct characteristics. It is divided by a web of narrow footpaths and surrounded by a tarmac road, buzzing with businesses, market stalls, and street kitchens. There is a youth group; a managed public water point; multiple churches and mosques, serving as community schools on weekdays; and numerous *chang'aa dens* (local bars where women distil and sell cheap home-brewed hard liquor).

### 2.2 Research team

We collected and analysed data from August 2020 to July 2021 (Supplementary Fig. 1). BR initiated and led the study, drawing on her background in social education, global health, GBV programmes, and HIV research. She designed the study with LVCT Health and formed a research team involving three ARISE-affiliated Kenyan researchers and 11 community co-researchers from Korogocho (Table 1) who met weekly face-to-face in Korogocho.

**Table 1:** Research team.

Domain	Community co-researchers (n = 11)	Researchers (n = 4)
**Initials**	AK, FN, JK, LM, MK, MM, MN, MW, NN, WL, ZO	BR, FM, MM, VM
**Backgrounds**		
Age (years)	Range: 21-56y Mean: 33y	Range: 30-45y Mean: 40y
Gender	8 women, 3 men	4 women
Education	1 Primary, 7 Secondary, 2 Post-secondary	1 Diploma, 2 Bachelor, 1 Master
Ethnicity	All Kenyan: 1 Borana, 1 Kamba, 6 Kikuyu, 1 Maasai, 1 Luhya, 1 Luo	1 German, 3 Kenyan
Religion	10 Christian, 1 Muslim	4 Christian
Residence	Korogocho	Formal settlements
Research experience	None: 3 Some (e.g., mobilisation, data collection, community advisory board): 8	Qualitative data collection: 4 Documenting, reporting: 4 Transcribing, translating: 3 Qualitative analysis: 4 Participatory research/work: 3
**Roles**		
Research team meetings	Participating, decision-making, planning, reviewing, reflecting	Facilitating, listening
Tools	Piloting, validating	Designing, updating
Sampling	Selecting participant groups, recruiting participants	Facilitating sampling, approaching key informants
Data collection	Co-facilitating, co-interviewing	Consenting, facilitating, interviewing
Data analysis	Reading, codebook development, summarizing data, analysing, translating	As co-researchers plus planning, facilitating, coding, documenting
Documentation	Taking photos	Note taking, report writing, photos
Dissemination	Planning, mobilising, moderating, presenting, evaluating	As co-researchers plus documenting

BR trained experienced researchers, including an initial 3-day training on participatory health research, ALIV[H]E, reflexivity, positionality, and power theories; and additional training in research ethics, safeguarding, conflict management, and academic writing. The researchers in turn facilitated and documented research team meetings and activities.

Community co-researchers were recruited through community health assistants and DREAMS staff to represent community volunteers and key populations (e.g., persons with disability). Since women bear the brunt of HIV and IPV, we selected greater representation of women, started with three male and five female co-researchers, and recruited two more in March 2021. A female lay translator took on additional roles and effectively became a co-researcher. When joining the project, co-researchers were 21–56 years old (Table 1), worked and/or lived in Gitathuru. Researchers trained co-researchers, with an initial 3-month period of group formation, problem definition and preparing data collection. Researchers debriefed after each activity and in monthly group counselling sessions. Co-researchers participated in sampling, data collection, analysis, and dissemination of findings to community stakeholders. Co-researchers’ time was compensated with a stipend (Ksh2,000/US$18 per month) and transport refund (Ksh500/US$4.50 per activity).

### 2.3 Participant sampling and recruitment

We were primarily interested in community views, engaged 56 women and 32 men in participatory workshops (Table 2) and interviewed ten key informants. We organised mixed-sex workshops with additional safety measures (outlined in 2.4 and 2.7) as women and men lacked opportunity in everyday life to talk with each other about sexual health. The geographical focus limited our ability to involve intersex, transgender, or non-binary persons.

**Table 2:** Characteristics of focus group participants.

Domain	Characteristics
Age (years)	Range: 18-72y Median (interquartile range): 26y (23-37.5y)
Gender	56 women 32 men
Education	None: 3 Primary: 28 Secondary: 37 Post-secondary: 11
Ethnicity	7 Kamba, 35 Kikuyu, 14 Luhya, 13 Luo, Other (6) (incl. Boran, Digo, Embu, Gabbra, Garre, Kisii, Meru, Swahili)
Religion	78 Christian, 10 Muslim
Characteristics as primary basis for recruitment for separate workshops	Female sex workers (n = 8) Men who have sex with men (n = 8) People living with HIV (n = 9) People who use drugs (n = 8) Persons with disability (n = 10, incl. hearing, physical or visual impairment) Women who have sex with women (8) Young people who married early (n = 10) Young women (n = 9) Community-based organisation (n = 10) Community health volunteers (n = 8)

We used a two-stage purposive sampling strategy for workshops. First, co-researchers prioritised key populations as a primary basis for recruitment for separate workshops (maximum variation sampling). Second, co-researchers identified potential participants from their social networks and by snowball sampling based on inclusion criteria (i.e., at least 18 years old; lives or works in Gitathuru; speaks English, Swahili, or Sheng). Co-researchers approached them face-to-face and gave invitation letters outlining research purpose and details. Key informants were selected based on their role in HIV and/or IPV prevention, including representatives from local administration (n = 2), law enforcement (n = 1), community-based organisations (n = 2), youth groups (n = 3), and facility-based HIV care (n = 2). They were approached face-to-face or via phone.

### 2.4 Data collection

We conducted workshops and key informant interviews (KIIs) between November 2020 and March 2021. Workshops and KII topic guides adapted ALIV[H]E guiding questions to focus on HIV and IPV, combined with group exercises during workshops. We piloted topic guides with co-researchers who confirmed relevance of questions and exercises. On their recommendation, we included the impact of COVID-19 on HIV and IPV and changed the flow of KII questions.

We held workshops at community halls in the Chief's camp. A researcher (VM or FM) facilitated workshops with two co-researchers (AK, JK, LM, MK, MM, MN, or WL). Sessions were conducted in Swahili and Sheng and lasted 2–4 h. Sheng is a mixed language which emerged in Nairobi's multilingual informal settlements. Facilitators introduced the study, obtained informed consent, and agreed group norms. Participants explored views on IPV and HIV using a spectrum line method (Priestley, 2015). They explored drivers of IPV and HIV by drawing spider diagrams (Loewenson et al., 2014) in single-gender groups to ensure women and men could speak freely. Afterwards, participants discussed identified drivers (spider legs) shaping IPV and HIV among women or men (spider's body) as well as the impact of COVID-19 control measures on IPV and HIV in a gender-mixed group. Workshops were closed with a breathing exercise for relaxation and self-affirmation. With participant consent, discussions were audio recorded and charts photographed. FM or an ARISE research assistant took notes in English. We added two additional workshops: (1) with the hearing impaired, engaging professional sign language interpreters, and (2) with *Lele* (women who have sex with women) after recruiting a *Lele-*identifying co-researcher to involve this hidden population.

BR and a co-researcher (FN, MK, or LM) interviewed key informants at their place of work or LVCT Health DREAMS site. Interviews were audio recorded, and BR took notes. Most interviews were in English and lasted 45–90 min. Co-researchers asked questions and aided with translation if interviewees preferred Swahili.

### 2.5 Analysis

We jointly analysed workshop data in multiple in-person workshops from February to June 2021. We used inductive and deductive approaches and incorporated established power theories and intersectionality frameworks in our analysis (Collins and Bilge, 2020; Hankivsky, 2014; Rowlands, 1997). We followed the collaborative data analysis model DEPICT, with six steps encompassing Dynamic reading, Engaged codebook development, Participatory coding, Inclusive reviewing and summarizing of categories, Collaborative analysing, and Translating (Flicker and Nixon, 2015): (1) We read workshop transcripts in pairs highlighting main issues on hard copies. (2) BR collated and clustered identified issues according to ALIV[H]E power domains and developed a draft codebook. Co-researchers validated the analytical framework, reviewed, rephrased, and added codes. (3) BR coded data manually and organised in MS Word tables. (4) Co-researchers summarised data code-by-code in small groups on flipcharts. BR and MM typed and managed summaries. Researchers complemented summaries using outsider perspectives. (5) We jointly evaluated data summaries, identified emerging issues, and agreed on cross-cutting themes. (6) We prioritised stakeholders and platforms, developed drama and artwork to disseminate findings. BR additionally analysed KII data. She tested and refined the existing codebook, then coded, extracted, and summarised data, comparing emerging themes with workshop data. KII data adding insights not covered in workshops were incorporated. BR, MT, and RT clustered themes, identified overarching power dynamics, and interpreted within the context. VM and MM presented further analysis to co-researchers who discussed and validated findings.

### 2.6 Quality assurance

Being a diverse research team of insiders and outsiders enabled us to incorporate multiple perspectives throughout the research process. We took steps to mitigate the effects of unequal power relations among and between participants, co-researchers, and researchers. We set and reviewed group norms, maintained a safe space, role-modelled core values such as respect for diversity, and involved co-researchers in decision-making. We used Swahili and Sheng, interactive facilitation techniques, visual methods, and group work to maximise participation and amplify ‘silent’ voices, embedding learning through regular group reflection on individual well-being, social positions, incomplete knowledge, power, and potential biases (Chowdhury et al., 2021). We considered emerging issues in our analysis and discussed unexpected findings, outliers, and silences in the data to ensure we do not neglect minority or critical voices. Co-researchers validated findings as did stakeholders in dissemination meetings. We used the participatory action research and COREQ checklists for reporting (Supplementary Table 1) and provide a structured reflexivity statement (Supplementary Table 2).

### 2.7 Ethics

The study was registered and approved by the Kenya National Commission for Science, Technology & Innovation (NACOSTI/P/19/1568 and NACOSTI/P/21/8210), AMREF Health Africa Ethics & Scientific Review Committee (P670/2019), and Liverpool School of Tropical Medicine Research Ethics Committee (19-065). Nairobi County and Ruaraka Sub-county Health Management Committees and local government authorities endorsed the study. We adopted evidence-based measures to mitigate risk of COVID-19. Participation was voluntary; co-researchers and participants gave written informed consent, including for audio recording. We discussed confidentiality repeatedly with co-researchers (e.g., initial one-on-one meetings, group reflections) and participants (e.g., recruitment, consent procedure). Although we diverted from WHO (2001) safety protocols by inviting women and men together, we ensured they were not from same households. To mitigate re-traumatisation and adverse events, co-researchers collected feedback from participants after workshops using a short questionnaire and informed participants about available counselling services.

## 3 Results

The people we spoke with were aware of HIV and IPV, ways of acquiring and preventing HIV, and relevant services. However, service providers reported HIV myths persisted alongside official information. Participants mainly understood IPV as physical violence and appreciated learning about emotional, economic, and sexual IPV, which they felt resonated with community realities. Though IPV was to some extent normalised, participants worried about intergenerational cycles of violence:

*“Children are affected by the fighting. When the parents fight, children think that violence is okay … When those children get married, they will also be abusive with their spouses,”* (W06, persons with disability).

**Fig. 2:**
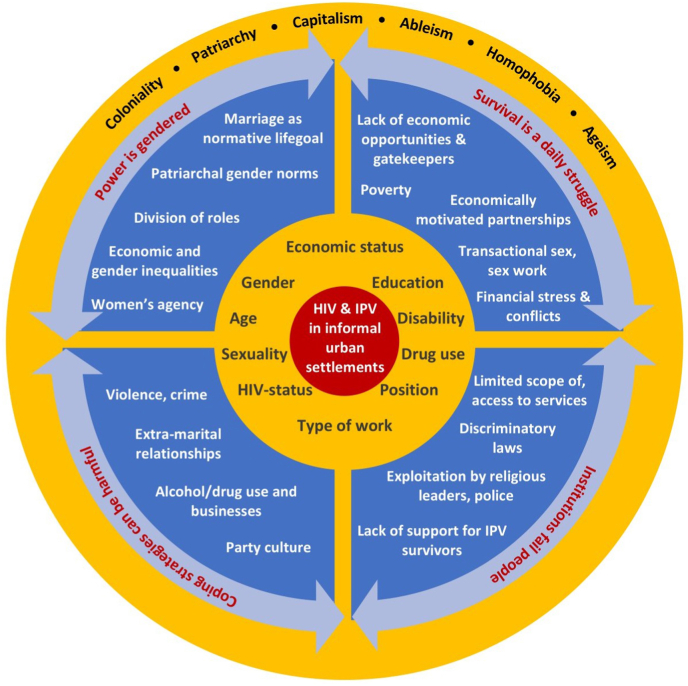
Intersectional processes of power linking HIV and IPV. Vulnerability to HIV and IPV shaped by processes of power and poverty (main themes shown in the arrows and segmented circle) influenced by interrelated structural forces of power (outer circle) and interconnected axes of power (second circle from inside).

Unequal power relations and the ways in which power was used “*wrongfully*” (co-researchers’ phrase) for material or symbolic benefit influenced vulnerability to IPV and HIV. Four central themes (shown in Fig. 2) summarise processes of power and poverty which we describe below. We found power domains in the initial conceptual framework – internalised values, access to resources, social norms, and policies – operated simultaneously. Experience and impact of poverty and power processes driving IPV and HIV were influenced by aspects of identity due to multiple, connected structural forces of power.

### 3.1 Theme 1: survival is a daily struggle

Vulnerability to IPV and HIV emerged through the daily struggles for cash and survival in the informal settlement. Experiences of poverty were frequent, including food insecurity and lack of money to pay rent, school fees, or medical bills. Narratives of financial hardship were ubiquitous but not uniform. Social marginalisation and gatekeepers played a critical role in alleviating, reinforcing, or aggravating social and material circumstances of individuals and social groups.

Opportunities for employment, income, and social protection were scarce. Many relied on daily income from casual labour, informal jobs, exploitative employment, and/or small businesses with low and unstable revenues for survival. Overall, men had greater chances to earn money in the nearby industries or dumpsite. Gendered division of roles limited women's economic participation. Whilst gatekeepers such as chiefs and village elders, mainly men, could select who accessed jobs, programmes, project handouts, and roles, NGOs and religious leaders also exercised power determining participation in programmes.

Community members were dissatisfied with unfair selection procedures and lack of accountability mechanisms especially for the social protection programmes introduced during the COVID-19 lockdown. Aid did not reach those groups identified by donors as most in need, leaving them more vulnerable. Persons with disability felt their abilities and needs were overlooked. They were underrepresented in unpaid and paid positions in the community and regularly missed out on education, employment, and social protection opportunities, even those meant for them.

*“When projects come, they ask for names and photos. When you look for your name later on, you won’t find it. You also pay for the form. We paid 150 (KSh/US$1.40) so the process is hurried. But in the end, all these names were removed,”* (W06, persons with disability).

Most women, but also some men, aspired to escape poverty and unemployment in the informal settlement through love relationships, transactional sex, and/or sex work, all of which were reported to trigger IPV and HIV exposure. Most same-sex and heterosexual relationships (including marriage) were financially motivated and transactional in nature.

“*It’s a contract. When the money stops coming in, you leave them*,” (W11, *Lele*)

*“If the wife doesn’t have sex with the husband, then he doesn’t give her money for upkeep”* (W02, young people who married early).

Pressures to find a financially stable partner created competition among young women for supposed ‘good husbands’ whilst pushing them into relationships with supposedly better-off men. Some men used their economic advantage to initiate sex and relationships with girls and young women through gifts like phones or money. Often, friends and parents put pressure on girls and young women to engage in intimate and transactional relationships, particularly on first-born daughters expected to take care of the family.

*“Some parents are pushing underage girls to go to a man’s house. This man in turn provides for the family. Especially in this COVID-era, a lot of girls will not go back to school because of this,”* (W03, young people who married early).

Financial disparities between partners created power imbalances as co-researchers explained: “*People with money get it their way*”. Within transactional, romantic, and marital relationships, partners could use their economic advantage to dictate partnership terms and control financially dependent partners, including through violence.

Exchanging sex for money, food, shelter, scholarships, transportation, or other basic needs offered brief relief from poverty, whilst increasing vulnerability to HIV and IPV. For survival, many women and men who have sex with men had transactional relationships with men, called *sponyo,* and some young men had sex with financially stable women, called *sugar mummies*. Financially dependent persons faced similar experiences of powerlessness and vulnerability to IPV and HIV due to the power their sexual partners wielded over them.

*“When a man gives you money to help your family, he will want to have sex without protection. If you refuse, he will beat you up,”* (W04, young women).

“*She (sugar mummy) has money, she provides you with everything. Because you are poor, you will feel powerless,*” (W03, young people who married early).

*“They (sponyo) are free to do what they want because they have money. You have to respect them to get money from them. They have a lot of power,”* (W05, men who have sex with men).

Although boundaries between transactional sex and sex work were blurred, relying on sex work as the main source of income heightened women's exposure to HIV and IPV. Male partners of female sex workers were reported to use violence to affirm authority, demand money or sex, or ban them from sex work.

*“A man may refuse to work, just use drugs and alcohol. When you work, get your own money, he takes the money. Then he still wants to sleep with you at night. And he does not want to provide for you. He wants to sleep with you by force,”* (W08, female sex workers).

Female sex workers' vulnerability to exploitation and violence increased during COVID-19 measures, when they met clients at their own or client's home due to restrictions (including bar closure and curfew). They also experienced stigma because community members believed children became sexually active if they observed sexual activities of sex workers in their congested neighbourhood during school closures.

Simultaneously, female sex workers stressed agency. They valued their income as a source of power and used female condoms to mitigate HIV exposure. *“If the client refuses to wear a condom, you can wear a female condom. By the time he finds out you are wearing one, he is done,”* (W08, female sex workers).

### 3.2 Theme 2: power is gendered

The financial power dynamics in the community, families, and relationships are innately linked with patriarchal gender norms and roles. Young people married and had children to meet social expectations of heterosexual marriage and family as a normative life goal. Their chances of finding a partner were mostly limited within Korogocho. Persons with disabilities, especially women, faced greater challenges in finding a partner affecting their ability to protect themselves from exposure to HIV and IPV. *“Because you feel no one will ever love you, you will be with whoever is available. Even if you don't know her status,”* (W06, persons with disability).

With little guidance and support to navigate marriage and family life, many young couples aspired to conform to patriarchal gender divisions of labour. Men, as heads of families, needed to provide financially, while women were responsible for domestic chores and childcare. Gender norms often condoned IPV, including as a way of showing love, as a young woman explained: *“If a man has not beaten you, you are not loved,”* (W03, young people who married early).

Intersecting gender and economic inequalities increased women's vulnerability to IPV and HIV. Married and unmarried women were reported to experience physical and sexual IPV frequently at the hands of their male partners. They were also vulnerable to HIV because of limited control over sex and use of condoms. Whilst the law provides protection against marital rape, patriarchal norms maintained a man's perceived right to sex within marriage.

*“You can be raped by your husband or boyfriend, and people will not believe you when you tell them. They will ask you, how can your husband rape you?”* (W06, persons with disability).

HIV diagnosis impacted women and men differently; women living with HIV felt particularly powerless in sero-different partnerships. HIV-negative partners could (threaten to) refuse sex, withdraw, separate, take children away, or disclose their HIV status to family and neighbours. According to community members and service providers, women tended to stay with a husband diagnosed with HIV, while men tended to divorce a wife diagnosed with HIV.

*“If the husband is negative, they could easily leave the wife [living with HIV] and marry another one” (W01, people living with HIV).*

Although marriage was perceived as a way out of poverty, the financial burden of sustaining a family put psychological pressure on couples. Stress, partnership conflict, and IPV chances were amplified during COVID-19 when widespread loss of income and jobs exacerbated poverty and food insecurity. *“Because there are no jobs, the husband is home, and he will stress the wife because he is also stressed because of lack of work,”* (W09, community-based organisation representatives). Conflicts also arose when men spent money on alcohol and extra-marital relationships.

Some women challenged the narrative of women's economic and social dependence on husbands. They contested notions of power as control, emphasising women's power-to or *“strength of a woman,”* (W11, *Lele*). *Lele* lived independently, as this woman stated, *“you only need a man when you need to make a baby,”* (W11, *Lele*). Women in heterosexual relationships hid money from partners and/or resorted to transactional relationships to take care of the children and themselves, running the risk of IPV and HIV exposure. Women who provided for the family could contest male dominance and influenced domestic decisions.

*“A man with no money has to listen to the wife or the mother if she is the one that provides for him. But a man who has money has the power over his house,” (W04, young women)*.

Roles and power relations in same-sex partnerships of women and men resembled those in heterosexual partnerships, involving a provider (sometimes called ‘father’ among *Lele*) and a dependent (sometimes called ‘wife’ among men). *Lele* reported IPV to happen rarely in *Lele* partnerships, perceived their chances of acquiring HIV to be low, and generally praised female partners for meeting each other's basic, emotional, and sexual desires. In contrast, financially dependent men who have sex with men felt vulnerable to IPV.

*“If you are there because of money, then there is no love there. That is why there is violence when conflict happens, because there's no love,”* (W05, men who have sex with men).

### 3.3 Theme 3: coping strategies may be harmful

Faced with limited options to mitigate the effects of poverty and powerlessness, many women and men resorted to coping strategies that put them, their partners and families in harm's way, whilst often reinforcing existing inequalities.

Young men, whose social influence and power were restrained due to their age, experienced unemployment and material deprivation as particularly disempowering. They reported experiencing emotional and physical IPV when they could not live up to gendered expectations.

*“Let’s say he did not find work but has looked the whole day. He comes home with no money. She wants money, but she does not want to hear the story. She will shout and even throw his things outside,”* (KI08, youth leader).

In response, some young heterosexual men resorted to violence, including IPV, as a means of affirming their masculinity and/or to theft or crime as means to gain income and status. This in turn put female partners in danger.

*“If a policeman knows my husband is a thief and always arrests him every time, then I will have sex with him so that he can stop arresting my husband,”* (W02, people who use drugs).

Patriarchal gender norms permitted men to have extra-marital relationships with a *mpango wa kando* (literally ‘side plan’ referring to an extra-marital sexual partner), including to demonstrate manhood. Married women tended to engage in *mpango wa kando* relationships when husbands didn't meet their emotional needs. Concurrent sexual partnerships increased chances of HIV exposure. Some young women reported using HIV pre-exposure prophylaxis, available from local HIV prevention projects, when they had extra-marital or transactional relationships or suspected partner's infidelity. Nonetheless, suspected or known *mpango wa kando* relationships of either partner could trigger conflict and IPV.

Many people used alcohol and/or drugs to cope with stress caused by poverty and other hardships. Alcohol and drugs were cheaply available in the informal settlement, albeit illegal and unregulated. Generally, young people preferred *bhangi* (marijuana), while middle-aged people consumed *chang'aa* (hard liquor). Many felt alcohol and drug use were common and normalised but also amplified vulnerability to HIV and IPV through aggression, condomless sex, or neglecting antiretroviral medication when intoxicated. Alcohol and drug businesses in the informal settlement increased vulnerability of young women. Men offered women drinks or drugs in exchange for sex – either agreed or forced – and paid commission to owners of *chang'aa dens*, called *Mama Pima*, who benefitted from transactional sex between their (young) female waiters and male customers. Male drug dealers used their power to demand sex from female and male consumers or their partners.

*“When you don’t have money, you sleep with them to get the drugs,”* (W03, young people who married early).

*“They (drug dealers) also have money, so you can call them when you need help. Either money or drugs. If you are addicted, you are under their control. If you don’t have money for the drugs, you have to sleep with them,”* (W05, men who have sex with men).

Alcohol, peer pressure, and norms entrenched in a party culture, called *bash*, exposed young people to HIV. Marital and dating relationships were reportedly suspended at a *bash* where partygoers could have sex with anybody. Although many young people felt *bash* rules were fair, the party culture caused financial stress and partner conflict, triggering IPV.

### 3.4 Theme 4: institutions fail people

Formal and informal institutions played a complex role in HIV and IPV response. Community members relied on them, but structural violence fuelled abuse and mistrust.

HIV and IPV-related prevention and empowerment programmes predominantly engaged adolescent girls and young women and largely excluded males. The few male-specific HIV and IPV outreach activities were suspended during COVID-19. Young women were better informed about HIV prevention than young men. Community members and stakeholders criticised the one-sided approach alike. They felt the lack of empowerment and economic opportunities for boys and young men in the informal settlement made them join gangs. Critical views ranged from a backlash among those worrying women's empowerment would make men lose power to considerations highlighting the limitations of one-sided empowerment: *“The same boys are the ones that will marry our girls and then the cycle will continue,”* (W07, community health volunteers). Furthermore, HIV and IPV response overlooked people with disability for whom vital information and services remained inaccessible.

Most women, who experienced IPV, sought support from informal institutions first before turning to formal institutions for help. Many times, help-seeking exposed women to greater harm. Families encouraged them to protect their marriage and stay with abusive partners. Neighbours and others were hesitant to intervene for fear of getting injured or accused of being the victim's *mpango wa kando*. Women who separated from (abusive) partners were stigmatised, “*when you decide to leave your marriage and stay single, you are called a prostitute,*” (W07, community health volunteers).

Women also turned to religious institutions for help. Pastoral and spiritual services could help couples overcome challenges and conflicts. More often, male religious leaders and traditional healers, called *Japolo*, failed women. Support from religious leaders could trigger IPV when husbands saw leaders as threats to their own authority. Several religious leaders and *Japolo* exposed followers to HIV by discouraging HIV testing and medication or demanding sex as therapy or fees.

*“Women really believe them (Japolo) so much to an extent whereby if a woman can’t conceive, the pastor can tell her that he will have sex with her as he prays for her so that she can conceive, and she agrees,”* (W02, people who use drugs).

Community members mistrusted the police and tended to report IPV to NGOs. NGOs were trusted more because they handled IPV with confidentiality and cases reported through NGOs were investigated more diligently by police. Policemen were feared because they could use their power to demand money or sex from complainants, accept bribes to release suspects, and arrest people.

*“Policemen are official sponsors. They have money and position. If they arrest you, you can sleep with them to be freed”* (W03, young people who married early).

Engagements by men who have sex with men, *Lele,* and sex workers with formal institutions were affected by laws and policies defining them as illegal or ‘key populations’. Many refrained from seeking help for IPV from local government and law enforcement agencies to avoid harassment, abuse, and extortion.

*“He(police officer)may laugh at you or harass you. You may be taken to counselling. Or they say they want to test your HIV status,”* (W05, men who have sex with men).

*“You also have to test for HIV. If you don’t agree to the test, they* (*healthcare providers at the key population clinic*) *will not help you”* (FGD08, female sex workers).

Overall, we observed gatekeepers recognised their lack of power within systems of power but not the privileges gained from the same. Similarly, community members who experienced inequalities themselves tended to be judgemental about the behaviour of others, unaware of barriers others faced.

## 4 Discussion

We combined the ALIV[H]E framework, intersectionality lens, and participatory methods to gain a nuanced understanding of the power dynamics in the informal urban settlement and unpick how these influence vulnerabilities to IPV and HIV. Our findings suggest the IPV and HIV nexus is rooted in the daily struggle for cash and survival in the informal settlement where lucrative livelihoods are scarce, power is used as control, a few gatekeepers regulate access to opportunities, and common coping strategies amplify exposure to HIV and IPV. Power relations of gender, economic status, age, ability, and/or sexuality work together in shaping inequities and driving IPV and HIV (Dunaway et al., 2022). Our study offers insights into the informal urban settlement context where women and men from key populations experience mutually reinforcing power imbalances having spiralling effects on the vulnerabilities underlying IPV and HIV.

Most people living in informal urban settlements do not achieve their constitutional rights and freedoms, including to food, shelter, health, and information among others (The Constitution Of Kenya, 2010). Failures to respect and protect the human rights of informal urban settlement communities give rise to IPV and HIV directly and indirectly through various other problems: poverty (SDG 1); food insecurity (SDG 2); lack of access to education (SDG 4); structural violence against women (SDG 5); poor living conditions (SDG 10); unemployment and exploitative employment (SDG 11); social injustices (SDG 16); and structural violence against HIV transmission, same-sex, drug use, and sex work. Given the complexity, bio-medical, disease specific, and siloed interventions are inadequate to deliver on the Government of Kenya's commitments to eliminate all forms of GBV by 2026 and to end HIV by 2030 (Government of Kenya, 2021; National AIDS Control Council, 2021). Plans towards the integration of HIV and GBV services (Ministry of Health, 2018) need to be complemented by multisectoral collaboration beyond health.

Structural, legal and interpersonal violence underpin HIV and IPV. Many formal and informal institutions do not engage informal urban settlement dwellers as rights holders. Instead, external, top-down programmes approach them as aid beneficiaries or as target groups for disease control, resonating with concerns women living with HIV raised (Dunaway et al., 2022). Failures to implement laws designed to protect women against violence lead to underreporting, low prosecution rates, and abuse and exploitation of women in private and public spheres (Committee on the Elimination of Discrimination against Women, 2017). Legal violence, “*the normalised but cumulatively injurious effects of the law*” (Menjívar and Abrego, 2012), and harmful social contexts induce interpersonal violence against men who have sex with men, *Lele,* people who use drugs, and sex workers (Kenya National Commission on Human Rights, 2011). Institutions and programmes must create safe places for women and key populations in informal urban settlements; uphold and protect human, sexual and reproductive rights of everyone; and address human rights violations in private and public realms.

Our findings suggest gender power relations are complex and dynamic. Many women are affected by the intersecting economic and gender inequalities, limiting their ability to exercise control over their own safety and sexuality (Jewkes et al., 2010). Patriarchal gender norms and ideals of masculinity can also be harmful to some men as normative behaviours and expectations increase their vulnerability to HIV and IPV (Heise and McGrory, 2016; Ratele, 2008). The community suggested interventions for boys and young men, alongside existing programmes for girls and young women (Saul et al., 2018). Elsewhere, working with women and men has proven to increase success of IPV prevention programmes (Jewkes et al., 2020). Observed intersectional gender power dynamics may require multiple interventions to improve health and well-being for women and men as evidence suggests multi-pronged interventions engaging numerous stakeholders and addressing multiple drivers are most effective to reduce IPV (Kerr-Wilson et al., 2020). Combinations of economic and social empowerment programmes for women and men, couple interventions, parenting programmes, and community activism, all of which emerged as effective or promising in reducing violence against women (Kerr-Wilson et al., 2020), may be acceptable and beneficial in informal urban settlements. Gender-transformative approaches increase success of IPV prevention programmes (Jewkes et al., 2020) and engagement in HIV services (Fleming et al., 2016; Leddy et al., 2021). Gender-transformative interventions with men cannot overlook, but must engage with men's experiences of poverty and unemployment and men's resistance to change (Ratele, 2015). Overall, programmes need to promote gender empowerment beyond individual behaviour change, facilitate collective change processes (Jewkes et al., 2020), and shift power imbalances at structural levels (Greig and Flood, 2023).

Our approach demonstrates people from diverse backgrounds can co-create safe spaces for dialogue on power. For example, during workshops, people with disabilities demanded space for self-representation and self-organisation; young men recognised the benefits of comprehensive health information; and young people realised the need for better partner communication. The principles of the ALIV[H]E approach guided our search for, and learning about, more participatory forms of research on IPV and HIV. Research and programmes must provide opportunities for participation and positive power following examples of self-organising and leadership by federations of ‘slum dwellers’ (Lines and Makau, 2018) and women living with HIV who generated knowledge and frameworks, including ALIV[H]E (Hale et al., 2018).

### 4.1 Strengths and limitations

Engaging women and men in heterosexual and same-sex partnerships enabled us to analyse diverse and interconnected inequities underlying HIV and IPV experiences. We describe distinct features of female and male HIV and IPV, while highlighting how their vulnerabilities reinforce each other and exacerbate women's exposure. Although we collected cross-sectional data, our long-term community engagement helped us consider emerging issues during analysis and seek clarifications during writeup. Our approach facilitated learning among researchers and co-researchers as well as participants who reported to have gained knowledge from workshops.

Our study had several limitations. Although our jointly developed IPV definition was broad, focusing on one form of violence appeared somewhat artificial because of relations between different forms of violence frequently discussed by participants. The onset of the pandemic disrupted community entry but also amplified inequalities we may not have observed otherwise. We held workshops and meetings in the community hall at the Chief's camp because of its central location, accessibility, and space. Participants from key populations, who avoided the location, needed reassurance about data protection and independence of our study from the Chief's office for their privacy and safety. We could not capture the views of refugees, people who are homeless, or male sex workers due to budget and time constraints. As participation is time-consuming, yet critical for research and data quality, we faced trade-offs between speed and participation. For example, co-researchers took on smaller roles in workshops because we could not provide training needed to facilitate group exercises beforehand. Since history and social structure across informal urban settlements vary, our geographical focus may be a limitation. Nonetheless, the intensive community involvement allowed us to explore depth, potentially enhancing transferability.

### 4.2 Conclusions

The structural forces operating in informal urban settlements create and sustain complex dynamics of power, poverty, gatekeeping, and marginalisation which are conducive to IPV and HIV. Thus, HIV and IPV prevention interventions require multisectoral collaboration, should engage women and men, combine social and economic empowerment, and must safeguard human rights, safety and participation of everyone.

## CRediT author statement

Beate Ringwald: Conceptualisation, Formal Analysis, Funding Acquisition, Investigation, Methodology, Project administration, Visualization, Writing – Original Draft Preparation, Writing Review and Editing; Miriam Taegtmeyer: Conceptualisation, Formal Analysis, Methodology, Supervision, Writing – Original Draft Preparation, Writing Review and Editing; Veronicah Mwania: Formal Analysis, Investigation, Methodology, Writing Review and Editing; Mary Muthoki: Formal Analysis, Investigation, Methodology, Writing Review and Editing; Faith Munyao: Investigation, Methodology, Writing Review and Editing; Lina Digolo: Conceptualisation, Methodology, Supervision, Writing Review and Editing; Lilian Otiso: Conceptualisation, Writing Review and Editing; Anne S. Wangui Ngunjiri: Supervision, Writing Review and Editing; Robinson N. Karuga: Supervision, Writing Review and Editing; Rachel Tolhurst: Conceptualisation, Formal Analysis, Funding Acquisition, Methodology, Supervision, Writing – Original Draft Preparation, Writing Review and Editing; Korogocho ALIV[H]E research team: Formal Analysis, Investigation, Validation.

## Declaration of competing interest

None.

## Data Availability

The data that has been used is confidential.
