# Supplementary material for: Power and poverty: A participatory study on the complexities of HIV and intimate partner violence in an informal urban settlement in Nairobi, Kenya
Source: Soc Sci Med. 2023 Nov;336:116247. doi: 10.1016/j.socscimed.2023.116247 (PMC10622644; doi:10.1016/j.socscimed.2023.116247)
Supplement: Multimedia component 1 [file mmc1.pdf]

Power and poverty: A participatory study on the complexities of HIV and intimate partner violence in an informal urban settlement in Nairobi, Kenya

Supplementary Material

Contents

Supplementary Figures 2

Supplementary Fig. 1. Study timeline 2

Supplementary Tables 3

Supplementary Table 1. Reporting standards 3

Supplementary Table 2. Structured reflexivity statement 9

References 12

Supplementary Figures

Supplementary Fig. 1. Study timeline

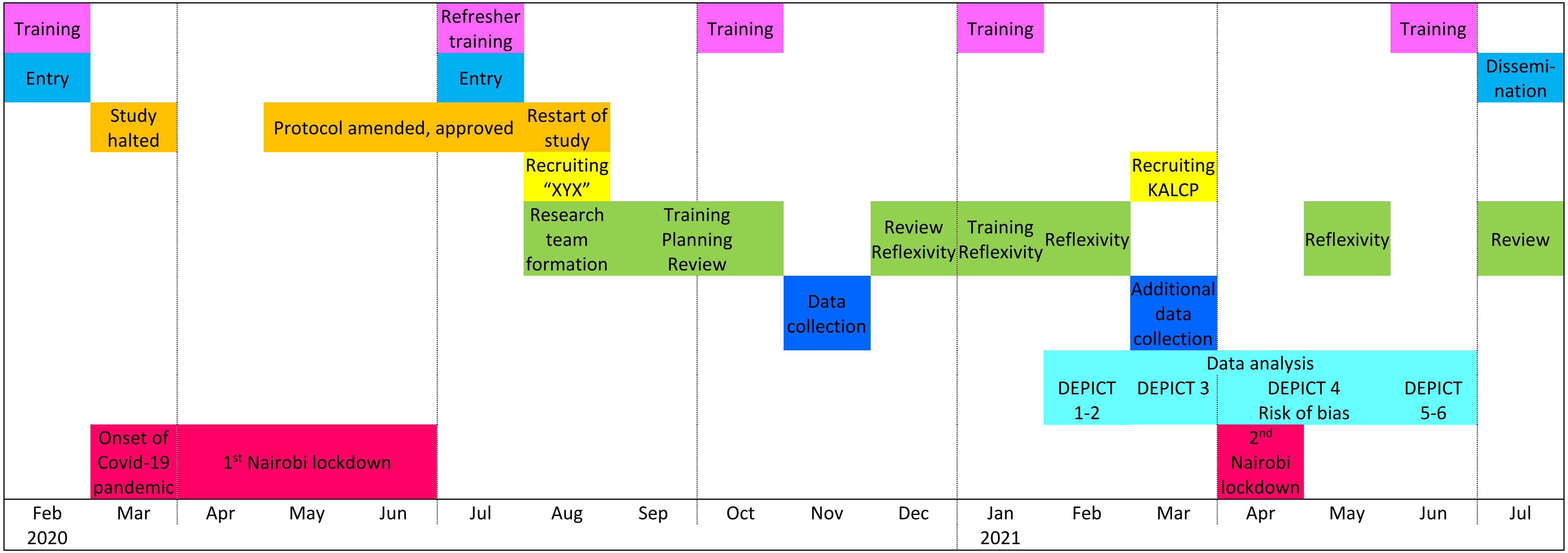

Note. Study timeline, exclusive of additional analysis from September 2021 to March 2022 and validation of findings in May 2022. Training = training of researchers. Entry = County, sub-county, and community entry. KALCP = Korogocho ALIV[H]E local community partners, the name community co-researchers chose for themselves. DEPICT = six-step participatory data analysis method.

Supplementary Tables

Supplementary Table 1. Reporting standards

Domain and question	Item No.	Author response	Reported on page
**Best Practices in the Reporting of Participatory Action Research (Smith et al., 2010)**			
**Domain 1: Key elements of the project**			
How was the project initiated?	1	The study was nested within ARISE, an international research consortium. BR initiated the project and designed the study with LVCT Health.	2,3
What was the project's timeframe?	2	1 year from August 2020 to July 2021 (Supplementary Fig. 1)	3
Who were the participants and/or co-researchers?	3	Co-researchers (n=11) selected to represent community volunteers and key populations (Table 1).<br>Workshop participants (n=88) selected to represent key populations (Table 2) and key informants (n=10)	3<br><br>3
What was the extent of their participation and nature of their roles?	4	Co-researchers were actively involved in the research process, including data collection, analysis, dissemination as outlined in Table 1.	4
What was the process within and/or the methodology of the project?	5	Participatory health research design<br>Focus group discussions and key informant interviews<br>Participatory data analysis (DEPICT)	2<br>4<br><br>4
What were the project outcomes and/or emergent actions?	6	Dissemination meetings with community stakeholders<br>Findings translated into community drama and artwork for continued dissemination and discussion	4
What comes next?	7		-
**Domain 2: Convey the Experiences of Co-Researchers**			
Pay attention to who is writing the article and how their voices and experiences are represented	8	BR discussed authorship with co-researchers. They suggested to include the "Korogocho ALIV[H]E research team" in the list of authors and to acknowledge their contribution as individuals in Acknowledgements.<br>Co-researchers validated findings.	8<br>4

Domain and question	Item No.	Author response	Reported on page
Pay attention to who is <u>not</u> writing the article and how their voices and experiences are represented	9	Co-researchers' role and contributions are explained in the article and acknowledged (see Acknowledgments).<br>Authors critically reflected on and discussed positionality, power and voice during analysis and writing to ensure fair presentation of the community.<br>Authors use illustrative quotes to include co-researcher and participant voice.	 <br>4<br><br>4-7
What were the personal outcomes of the project?	10	Increased knowledge and awareness among participants and co-researchers	8
**Domain 3: Address the Challenges, Pitfalls, and Limitations of the Project**			
What were they?	11	Participation and power relations within a diverse research team<br>Participation and power relations during gender-mixed workshops, including risk of re-traumatisation or harm<br>Within available time and funding some key populations were not reached	4<br>4<br>8
How were they managed?	12	Agreed group norms<br>Maintained a safe space<br>Role-modelled core values<br>Involved co-researchers in decision-making<br>Communicated in Swahili and Sheng<br>Used interactive facilitation techniques, visual methods, and group work<br>Embedded learning through regular group reflection<br>Conducted follow-up interviews with workshop participants<br>Provided information on IPV services	4
What can we learn?	13	Our participatory approach demonstrates people from diverse backgrounds can co-create safe spaces for dialogue on power.<br>Our participatory research design facilitated learning among researchers and co-researchers as well as participants who reported to have gained knowledge from workshops.<br>Intersectional approach, involving different key populations, enabled us to illustrate complex and fluid power dynamics involving multiple	8<br>8<br>4-7

Domain and question	Item No.	Author response	Reported on page
		intersecting axes of power that drive IPV and HIV in the informal urban settlement.	
**COnsolidated criteria for REporting Qualitative research (COREQ) Checklist (Tong et al., 2007)**			
**Domain 4: Research team and reflexivity**			
Personal characteristics			
Which author/s conducted the interview or focus group?	14	A researcher (VM or FM) facilitated worshops with two co-researchers (AK, JK, LM, MK, MM, MN, or WL).<br>BR and a co-researcher (FN, MK, or LM) interviewed key informants	4
What were the researcher's credentials?	15	Researchers: 1 Diploma, 2 Bachelor, 1 Master (see Table 1)<br>Co-researchers: 1 Primary, 7 Secondary, 2 Post-secondary education (see Table 1)	3
What was their occupation at the time of the study?	16	3 professional researchers, 1 PGR student (see Table 1)	3
Was the researcher male or female?	17	Researchers: 4 women<br>Co-researchers: 8 women, 3 men	3
What experience or training did the researcher have?	18	All researchers had skills in qualitative and quantitative research methods; 3 researchers had conducted participatory research or work (see Table 1).<br>Some co-researchers had been involved in research before (see Table 1).	3
Relationship with participants			
Was a relationship established prior to study commencement?	19	Co-researchers were known to LVCT Health. Relationships between researchers and co-researchers were established during the 3-month team formation and capacity strengthening phase that preceded data collection (see Supplementary Fig. 1).<br>Participants were known to co-researchers prior to the study.	3<br>4
What did the participants know about the researcher?	20	Participants were given invitation letters outlining research purpose and details.	4
What characteristics were reported about	21	Table 1 shows age, gender, education, ethnicity, religion, residence, and research experience of	3

Domain and question	Item No.	Author response	Reported on page
the interviewer/facilitator?		researchers and co-researchers. We report more background about BR who led the study.	
**Domain 5: Study design**			
Theoretical framework			
What methodological orientation was stated to underpin the study?	22	Conceptual framework (Fig. 1) incorporates intersectionality and ALIV[H]E power concepts<br>Participatory health research approach	2<br><br>2
Participant selection			
How were participants selected?	23	Two-stage purposive sampling strategy: (1) prioritising key populations as a primary basis for recruitment (maximum variation sampling); and (2) snowball sampling based on inclusion criteria.	4
How were participants approached?	24	Face-to-face, invitation letters given	4
How many participants were in the study?	25	Workshops: 56 women and 32 men<br>KII: 10 key informants	4
How many people refused to participate or dropped out?	26	None	-
Setting			
Where was the data collected?	27	Workshops: community halls in the chief's camp<br>KII: workplace or LVCT Health DREAMS site.	4
Was anyone else present besides the participants and researchers?	28	Only named researchers and co-researchers were present as outlined.	4
What are the important characteristics of the sample?	29	Characteristics of the sample are shown in Table 2, including attributes that served as primary basis for recruitment for separate workshops (i.e., men who have sex with men, people living with HIV, people who use drugs, persons with disability, women who have sex with women, young people who married early, young women, community-based organisation, community health volunteers).	3
Data collection			
Were questions, prompts, guides provided by the authors? Was it pilot tested?	30	Workshop and KII topic guides adapted ALIV[H]E questionnaires to focus on HIV and IPV.<br>Workshop questions were combined with group exercises.<br>Topic guides were piloted with co-researchers.	4

Domain and question	Item No.	Author response	Reported on page
Were repeat interviews carried out?	31	We added two additional workshops as outlined.	4
Did the research use audio or visual recording to collect the data?	32	Workshops were audio recorded and charts photographed. KII were audio recorded.	4
Were field notes made during and/or after the interview or focus group?	33	Notes were taken during workshops and KIIs.	4
What was the duration of the inter views or focus group?	34	Workshops: 2-4 hours<br>KII: 45-90 minutes	4
Was data saturation discussed?	35	We explain how we considered new and emerging issues in our participatory data analysis.<br>Co-researchers validated data and findings as did stakeholders in dissemination meetings.	4
Were transcripts returned to participants for comment and/or correction?	36	No. But co-researchers validated transcripts and findings. Community stakeholders validated findings during dissemination.	4
**Domain 6: analysis and findings**			
Data analysis			
How many data coders coded the data?	37	BR	4
Did authors provide a description of the coding tree?	38	Emerging codes were clustered according to ALIV[H]E power domains. Co-researchers reviewed and validated the codebook.	4
Were themes identified in advance or derived from the data?	39	The research team identified issues emerging from data summaries and agreed cross-cutting themes. BR, MT, and RT clustered themes, identified overarching power dynamics, and interpreted within the context.	4
What software, if applicable, was used to manage the data?	40	MS Word	4
Did participants provide feedback on the findings?	41	Community stakeholders validated findings during dissemination meetings (some of whom were interviewed).<br>Co-researchers discussed and validated findings generated by additional analysis.	4

**Domain and question**	**Item No.**	**Author response**	**Reported on page**
Reporting			
Were participant quotations presented to illustrate the themes/findings? Was each quotation identified?	42	We included direct quotes from participants. These are numbered, showing the primary characteristic of workshops.	4-7
Was there consistency between the data presented and the findings?	43	We provide illustrative quotes that relate to presented findings.	4-7
Were major themes clearly presented in the findings?	44	We structured findings according to 4 main themes and visualise in Fig. 2.	4-7
Is there a description of diverse cases or discussion of minor themes?	45	Minor themes are aligned to major themes as outlined in Fig. 2. We illustrate the distinct features of female and male HIV and IPV, considering how gender intersects with other axes of power. We outline similarities and differences between IPV and HIV within heterosexual and same-sex relationships.	4-7

Supplementary Table 2. Structured reflexivity statement

Our structured reflexivity statement is based on domains and questions proposed in the equitable authorship consensus statement" developed by Ben Morton and colleagues (2021).

Domains & Questions	Item No.	Author response	Reported on page
**Study conceptualisation**			
How does this study address local research and policy priorities?	1	The Government of Kenya committed eliminate all forms of gender-based violence (GBV) by 2026 (SDG 5) (National AIDS Control Council, 2021) and to end HIV by 2030 (SDG 3) (Government of Kenya, 2021). Kenyan health policies and frameworks seek to combine HIV and GBV prevention (National AIDS Control Council, 2021) and integrate HIV and GBV services (Ministry of Health, 2018).	7
How were local researchers involved in study design?	2	The study was nested within ARISE, an international research consortium. BR initiated the project and designed the study with LVCT Health.	3
**Research management**			
How has funding been used to support the local research team(s)?	3	Funding was used to support Kenyan members of the research team in the following ways:<br>• Professional fees and transport refund for researchers<br>• Stipends and transport refund for community co-researchers<br>• Training of researchers and co-researchers<br>• Psycho-social counselling for researchers and co-researchers	3-4
**Data acquisition and analysis**			
How are research staff who conducted data collection acknowledged?	4	• Kenyan researchers are co-authors.<br>• Community co-researchers are acknowledged for their contribution (see #10).	3<br>8-9
How have members of the research partnership been provided with access to study data?	5	• Main themes of study findings translated in paintings by local artist. Dissemination toolkit produced for community co-researchers (copy of paintings, guiding questions).<br>• Data shared with LVCT Health.	4
How were data used to develop analytical skills	6	Kenyan researchers and co-researchers were trained in participatory data analysis approach DEPICT (Flicker & Nixon, 2015). Data were analysed jointly.	3<br><br>4

within the partnership?			
**Data interpretation**			
How have research partners collaborated in interpreting study data?	7	BR, MT, and RT clustered themes, identified overarching power dynamics, and interpreted within the context. VM and MM presented further analysis to co-researchers who discussed and validated findings.	4
**Drafting and revising for intellectual content**			
How were research partners supported to develop writing skills?	8	BR trained researchers (MM and VM) in writing skills training. BR, FM, MM, and VM wrote and published several blogs together during the study (Kerubo et al., 2020; Munyao et al., 2020; Muthoki et al., 2022).	7
How will research products be shared to address local needs?	9	In July 2021, the research team disseminated study findings to (1) LVCT Health and (2) community stakeholders (including local government, law enforcement, health care providers, NGOs, and youth groups). Dissemination tool kits were developed to facilitate discussion of study findings in the community (see #5).	-
**Authorship**			
How is the leadership, contribution, and ownership of this work by LMIC researchers recognised within the authorship?	10	BR discussed authorship with the research team:<br>• Community co-researchers suggested to include the "Korogocho ALIV[H]E research team" in the list of authors and to acknowledge their contribution as individuals. The eleven co-researchers are recognised by name in Acknowledgements.<br>• Kenyan researchers involved in data collection and analysis are co-authors of the article (Authors 3-5).<br>• Kenyan senior researchers who supported the study as supervisors are co-authors (Authors 6-9).	8-9<br>4,8<br>8
How have early career researchers across the partnership been included within the authorship team?	11	The first author is an early career researcher. She conducted the research within a doctoral training programme and wrote the first draft.	3,8
How has gender balance been addressed within the authorship?	12	This is a female-led research project. More women than men were involved in the study as participants and co-researchers because of the gendered nature of the research topic. Gender ratio of authors reflects gender ratio of researchers.	3

**Training**			
How has the project contributed to training of LMIC researchers?	13	The study used a participatory capacity strengthening approach, building on existing knowledge and skills. Kenyan researchers gained knowledge and skills in participatory research approaches and methods; safeguarding; facilitation skills; power and intersectionality theories. Co-researchers gained knowledge and skills in participatory research, data collection and analysis; communication and visualisation skills.	3,4,8
**Infrastructure**			
How has the project contributed to improvements in local infrastructure?	14	N/A	-
**Governance**			
What safeguarding procedures were used to protect local study participants and researchers?	15	The study complied with ARISE safeguarding protocols (Aktar et al., 2020). A study safety and security plan was developed and followed. Specific measures include:<br>• Set and reviewed group norms<br>• Defined characteristics of safe space<br>• Discussed confidentiality at various time points<br>• Followed WHO safety measures<br>• Conducted gender-specific group work during workshops<br>• Followed up of workshop participants<br>• Provided participants with information on service providers and tollfree hotline<br>• Psycho-social counselling services	4

References

Aktar, B., Alam, W., Ali, S., Awal, A., Bayoh, M., Chumo, I., Contay, Y., Conteh, A., Dean, L., Dobson, S., Edstrom, J., Elsey, H., Farnaz, N., Garimella, S., Gray, L., Gupte, J., Hawkins, K., Hollihead, B., Josyula, K. L., . . . Wurie, H. (2020). How to prevent and address safeguarding concerns in global health research programmes: practice, process and positionality in marginalised spaces. *BMJ Global Health*, *5*(5), e002253. https://doi.org/10.1136/bmjgh-2019-002253

Flicker, S., & Nixon, S. A. (2015). The DEPICT model for participatory qualitative health promotion research analysis piloted in Canada, Zambia and South Africa. *Health Promot Int*, *30*(3), 616-624. https://doi.org/10.1093/heapro/dat093

Government of Kenya. (2021). *Generation Equality Forum: Kenya's Roadmap for Advancing Gender Equality and Ending all Forms of Gender Based Violence and Female Genital Mutilation by 2026*. https://www.genderinkenya.org/publication/generation-equality-forum-kenyas-roadmap-for-gender-equality/

Kerubo, M., Munyao, F., Muturi, J., Mwania, V., Ngoya, Y., Ringwald, B., & Wairimu, G. (2020). *Building partnerships for research*. https://lvcthealth.org/archives/4955

Ministry of Health. (2018). *National Sexual Reproductive Health and Rights, HIV, Sexual Gender Based Violence and Tuberculosis Integration Framework 2018-2022*.

Morton, B., Vercueil, A., Masekela, R., Heinz, E., Reimer, L., Saleh, S., Kalinga, C., Seekles, M., Biccard, B., Chakaya, J., Abimbola, S., Obasi, A., & Oriyo, N. (2021). Consensus statement on measures to promote equitable authorship in the publication of research from international partnerships. *Anaesthesia*, *77*(3), 264-276. https://doi.org/https://doi.org/10.1111/anae.15597

Munyao, F., Mwania, V., & Ringwald, B. (2020). *"It puts value on the people" Community Based Participatory Research in Kenya*. https://www.ariseconsortium.org/it-puts-value-on-the-people-community-based-participatory-research-in-kenya/

Muthoki, M., Mwania, V., & Ringwald, B. (2022). *A research journey that brought power theories to life: Lessons from Korogocho, Kenya*. https://www.ariseconsortium.org/a-research-journey-that-brought-power-theories-to-life-lessons-from-korogocho-kenya/

National AIDS Control Council. (2021). *Kenya AIDS Strategic Framework II - 2020/21-2024/25. Sustain Gains, Bridge Gaps and Accelerate Progress*. https://nacc.or.ke/kenya-aids-strategic-framework-kasf/

Smith, L., Rosenzweig, L., & Schmidt, M. (2010). Best Practices in the Reporting of Participatory Action Research: Embracing Both the Forest and the Trees. *Couns Psychol*, *38*(8), 1115-1138. https://doi.org/10.1177/0011000010376416

Tong, A., Sainsbury, P., & Craig, J. (2007). Consolidated criteria for reporting qualitative research (COREQ): a 32-item checklist for interviews and focus groups. *Int J Qual Health Care*, *19*(6), 349-357. https://doi.org/10.1093/intqhc/mzm042
